# Impact of Extracorporeal Shock Wave Lithotripsy (ESWL) on Kidney Length and Corticomedullary Differentiation in Patients With Renal Stones: A Case-Control Study

**DOI:** 10.7759/cureus.69760

**Published:** 2024-09-19

**Authors:** Zuhal Y Ahmed, Ahmed Abdelrahim, Awadia Gareeballah, Moawia Gameraddin, Maisa Elzaki, Shima I Ali, Mohammed A Hassan, Marwa H Mohammed, Raga A Abouraida

**Affiliations:** 1 Department of Diagnostic Radiologic Technology, Faculty of Radiology Science and Medical Imaging, Alzaiem Alazhari University, Khartoum, SDN; 2 Department of Diagnostic Radiology, College of Applied Medical Sciences, Taibah University, Al-Madinah Al-Munawwarah, SAU; 3 Faculty of Health Sciences, Gulf Medical University, Ajman, ARE; 4 Department of Radiological Sciences,College of Applied Medical Sciences, King Khalid University, Abha, SAU

**Keywords:** cmd, eswl, hydronephrosis, kidney length, renal stones

## Abstract

Background

The effect of extracorporeal shock wave lithotripsy (ESWL) on kidney morphology was evaluated sonographically in patients with renal stones. This study aimed to investigate the effects of ESWL on the kidneys after consecutive sessions.

Methods

This case-control study included adult participants from December 2018 to August 2022 in three major centers of ESWL treatment. Data were collected from 392 patients (336 treated with ESWL and 56 in the control group). Data were analyzed using SPSS Statistics (IBM Corp., Armonk, USA). Both binary logistic regression and generalized linear regression were applied to determine the factors that predicted the effect of ESWL on kidney length (KL) and cortico-medullary differentiation (CMD).

Results

The overall effect of ESWL treatment on patients with renal stones was observed in 19.9% disturbed CMD, and 11.6% decreased parenchymal thickness. KL was significantly decreased in patients exposed to ESWL compared to controls (9.103 vs.10.291 cm, p-value < .001). KL decreased significantly as the number of ESWL sessions increased (r = -.209, p-value < .001). After adjustment, the frequencies of ESWL and hydronephrosis were significantly associated with CMD distortion. Patients who were exposed to more than one ESWL session had 2.64 increased odds of distorted CMD as compared to controls (AOR=2.64, 95% CI = 1.040-6.683), and those with hydronephrosis had 1.70 increased odds of distorted CMD as compared to controls (AOR=1.70, 95% CI = 1.188-2.434).

Conclusion

ESWL significantly affected the renal length and CMD in patients with renal stones. The frequency of ESWL sessions significantly decreased KL and disturbed CMD in patients with renal stones. The outcome was not affected by the number of stones.

## Introduction

Extracorporeal shock wave lithotripsy (ESWL) has become a major treatment modality for symptomatic patients with renal stones [1]. The introduction of ESWL has revolutionized urolithiasis management as a treatment for urinary tract stones, spreading worldwide.

Since the indications for ESWL have been extended, 80% to 90% of calculi can now be successfully treated with ESWL. ESWL is still recommended as initial therapy for renal calculi up to 30 mm in diameter [2, 3].

However, the impact of ESWL on the kidneys has not been extensively studied. As lithotripsy is the only non-invasive treatment for urinary stones, SWL (Shock Wave Lithotripsy) is especially appealing because it can break most stone types. The kidneys and nearby tissues may experience vascular trauma owing to SWL. This acute SW damage has been associated with potentially significant long-term adverse effects, is potentially severe, can result in scarring and permanent renal loss, and can affect renal volume. The immediate effect of SWL on the kidney has been studied earlier and has been reported to have a significant effect on the function and morphology of the kidneys [4]. ESWL has been reported to cause tissue damage, which is most frequently traumatized, although it may injure the surrounding organs. Understandably, kidney damage has received much more attention than extrarenal effects [5]. Comprehensive morphological studies in various animals have demonstrated that SWL can damage the surrounding renal tubules and rupture the blood vessels. [6-9] Furthermore, several studies have documented mild-to-severe hemorrhages due to lithotripsy [6,7]. Several serious intraparenchymal, subcapsular, or perirenal bleeding consequences have been detailed in case reports, including irreversible acute renal failure [10-15]. Interestingly, serious complications can develop even when there is little bleeding. An example is irreversible acute renal failure in a person with anti-glomerular basement membrane disease, most likely due to glomerular injury during SWL [16].

The consequences of these findings may affect the renal size and distort the CMD.

To the best of our knowledge, no previous study has examined the effects of the frequency of ESWL on kidney length (KL) and corticomedullary differentiation (CMD) in the Sudanese population. There is less previous evidence for the late effects of ESWL on the kidneys. Therefore, this study aimed to determine whether ESWL has an effect after regular sessions of SWL treatment for a year.

## Materials and methods

Study design and data source

This cross-sectional case-control study deals with ultrasound findings of post-ESWL renal stone patients conducted in Sudan in the following hospital departments: Ultrasound and Lithotripsy, Mawada Hospital, Modern Medical Center & Alneilin Hospital, from December 2018 to August 2022.

Study population

The study included patients whose ages ranged from 9-80 years old, totaling 336 individuals. The control group was 56 healthy volunteers. The selected group of patients were those with one or more ESWL sessions on a regular follow-up basis and were scanned by ultrasound for close monitoring. Patients with associated diseases that could alter the size or CMD, such as diabetes, hypertension, and renal vascular diseases, were excluded from the study. The KL and CMD were both measured in patients and controls.

The sample size was obtained using the formula: n= N/ 1+N (e2), where n= sample size, N= the total population obtained from the hospitals, e= tolerable error (5%), the number of patients per month approximately 35, the number of the patients for 40 months 1400, the total sample n≅ 311 patients, in this study 336 patients were gathered. Data were collected using a data collection sheet specifically designed for this study. The control group was selected on the basis of the absence of diseases affecting the renal system. All the controls were asymptomatic at the time of the study.

Lithotripsy procedure

In all patients, the lithotripsy session was performed by Allengers UROLITH+, an extracorporeal shock wave lithotripsy equipment (Allengers Medical Systems Ltd., Chandigarh, India). The fragmentation laser system was configured to use 0.8 an of energy 8 Hertz. According to the user handbook, a maximum of 3,000 shocks were administered to patients with renal stones and a maximum of 4,000 shocks were administered to patients with ureteral stones. When remnant fragments of ≥ 4 mm were detected, ESWL was deemed unsuccessful. Patients who underwent an initial ESWL treatment failed to undergo a second ESWL treatment.

Sonographic procedures

An Esaote ultrasound machine (Esaote SpA, Genoa, Italy) with a multi-frequency curvilinear probe (3.5 - 5 MHz), variable focal zone and frequency capability, and Mindray DC-8 (Mindray, Shenzen, China) with two curvilinear probes (3.5 MHz). After properly setting the overall gain (system) gain and time gain or depth gain compensation (TGC), each patient was examined by the researcher and consultant radiologists/nephrologists to confirm the findings and diagnosis following the international scan guidelines and protocols. US examinations were performed on all patients 4 weeks after ESWL treatment. Patients with fever or excruciating pain from urinary tract infection were excluded.

The Sonographic Procedure of the Kidneys

Overlying bowel gas may obstruct abdominal scanning by causing total ultrasound reflection. The patient was examined after fasting, imposing dietary restrictions (avoidance of gas-producing foods), physical exercise, and water contrast to avoid this problem. The examination was performed with the patient in the supine position. Additional lateral decubitus and prone part scans are necessary and valuable in some situations, especially in obese patients or patients with skeletal deformations.

The examination of the kidneys was started with the patient in the supine position. Scans were performed in the sagittal and transverse planes from the anterior approach, using the liver and spleen as acoustic windows. Different movements for scanning the patients, such as left lateral decubitus and lateral oblique positions for the right kidney, and right lateral decubitus or lateral oblique positions for the left kidney, may improve the display of the kidneys. The kidney length was measured at the longest longitudinal diameter (bipolar axis). CMD was determined and qualitatively evaluated as preserved (normal) or disturbed based on distinguishing the sonographic appearance of the cortex and medulla.

Statistical analysis

Data were analyzed using SPSS Statistics version 23 (IBM Corp., Armonk, USA). Descriptive statistics were calculated for every measured variable, and frequencies and percentages were calculated for categorical data. The chi-squared test was then performed to assess the correlation between the study variables. Pearson correlation and regression analysis were performed to assess the impact of age and the number of lithotripsy sessions on kidney length, which was considered significant (P-value < 0.05), and an independent sample t-test was used to compare the kidney length in patients with lithotripsy and the control group.

## Results

A total of 336 patients underwent ESWL for the treatment of renal stones. Most of the patients were in the age groups of 18-38 years (51.1%) and 39-59 years (35.7%); 61.3% were males, and 38.7% were females (Table 1). 

**Table 1:** Demographic characteristics of the study population

Age	Frequency	Percent %
Age groups/years		
9-17	9	2.7
18-38	174	51.8
39-59	120	35.7
60-80	33	9.8
Gender		
Males	206	61.3
Females	130	38.7

The majority of the renal stones were solitary (59.5%), two stones (22.6%) and multiple stones were less frequent (17.9%). Most of the stone lengths ranged from 0.3-1 cm (66.4%) to 1.1-2 cm (29.5%), and their measurements > 2 cm were less frequent (4.2%). Most of these stones were found in the left kidney (47.9%), 46.4% in the right kidney, and 5.7% bilateral. Most of these stones were located in the lower calyces (56.5%) and 29.8% in the middle calyces (Table 2). Hydronephrosis was absent in 69% of the patients, while mild hydronephrosis was present in 19%, and 11.9% had moderate hydronephrosis (Table 2).

**Table 2:** Charecterisics and associated features of the renal stones in the study sample

Variables	frequency	Percent%
length of stones		
0.3-1 cm	223	66.4
1.1-2 cm	99	29.5
> 2 cm	14	4.2
Number of stones		
solitary	200	59.5
Two	76	22.6
Multiple	60	17.9
Laterality		
Right	156	46.4
Left	161	47.9
Bilateral	19	5.7
Stone location		
Upper calyx	46	13.7
Middle calyx	100	29.8
Lower calyx	190	56.5
Hydronephrosis		
None	232	69.0
Mild	64	19.0
Moderate	40	11.9

Kidney changes due to ESWL exposure are summarized in Table 3. It was observed that 80.1% and 88.1% had normal corticomedullary differentiation (CMD) and normal parenchymal thickness, respectively. The majority (89%) of patients were exposed to one to four sessions of ESWL for treating renal stones, while 10.4% were exposed to more than five sessions of ESWL. A significant linear correlation was observed between ESWL sessions, kidney length, and CMD (Table 3).

**Table 3:** Changes in the kidneys as a result of ESWL exposure ESWL: extracorporeal shock wave lithotripsy

changes	Frequency	Percent %
Corticomedullar differentiation		
Preserved	269	80.1
Disturbed	67	19.9
Parenchymal thickness		
Normal	296	88.1
Increased	1	.3
Decreased	39	11.6
Number of ESWL sessions		
1-4	299	89.0
5-8	35	10.4
9-11	2	.6

Kidney length decreased significantly as SWL sessions increased (r = -.209, P-value < 0.001), while CMD was disturbed in the same increased SWL sessions (r=.198, P-value < 0.001). Both renal length and CMD were affected by increased ESWL sessions. This influence was more evident in the comparison of kidney length between the control and ESWL exposure groups (Table 4).

**Table 4:** Correlations of ESWL sessions with kidney length and CMD in patients’ renal stones **Correlation is significant at the 0.01 level (2-tailed) A significant correlation was noticed between the number of ESWL, with both renal length and decreased CMD, both of them decreased as number of ESWL increased , p < 0.001. ESWL: extracorporeal shock wave lithotripsy; CMD: corticomedullary differentiation

Variables	Correlation coefficient	P-value
Kidney length	-.209^**^	< 0.001
CMD	.198^**^	< 0.001

The mean renal length was significantly lower in the exposure group than in the control group (9.1034 cm vs. 10.2911 cm, p < 0.001) (Table 5).

**Table 5:** Comparison of kidney length between the controls and ESWL exposure group Independant sample t-test demonstrated a significant difference in mean renal length between the ESWL exposure group and the control group, p < 0.001 ESWL: extracorporeal shock wave lithotripsy

Groups	N	Mean (cm)	Std. Deviation	P-value
ESWL group	336	9.103	.56002	< 0.001
Control group	56	10.291	.82622	

**Figure 1:**
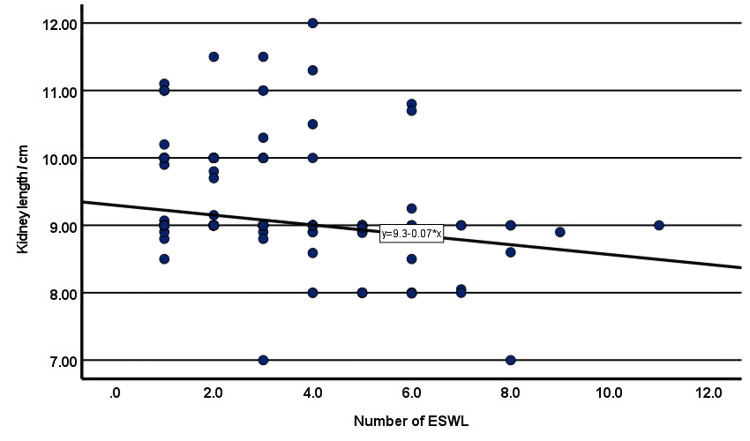
Regression estimate curve shows negative linear relationship between number of ESWL and kidney length in patients with renal stones ESWL: Extracorporeal Shock Wave Lithotripsy

The linear regression model predicted the renal length as an effect of increased SWL sessions. The kidney length decreases as number of ESWL session increased, the regression equation showed that each additional ESWL session was associated with a 0.07 cm decrease in kidney length (Figure 1).

The linear regression model in Table 6 clarifies the effect of ESWL sessions on KL, combined with patient age. The regression model showed that both had a significant effect on kidney length. The significance of ESWL sessions in KL was more robust than that of age (p < 0.001). The generated regression equation  revealed that each additional ESWL session was associated with a decrease of 0.074 cm in kidney length at .006 months (Table 6).

**Table 6:** Linear regression analysis for prediction of changes in kidney length in patients exposed to ESWL for treatment of renal stones Linear regression model of the effect of ESWL sessions on KL, combined with patient age, showed that both had a significant effect on kidney length  (p < 0.001, 0.009 for number of ESWL and age, respectively). ESWL: Extracorporeal Shock Wave Lithotripsy; KL: kidney length

Variables	Unstandardized Coefficients		Significance P-value	95% CI	Correlation coefficient
	B	Standard error			
The age	-.074	.002	.009	.001–.010	.13*
Number of ESWL sessions	.006	.019	< .001	-.111– -.038	-.21**
Constant	9.079	.102	< .001	8.879 – 9.280	
Regression equation	KL=9.079 + .006 x age -.074 x SWL sessions				
.					

Logistic regression analysis for the prediction of CMD disturbance in patients exposed to ESWL for the treatment of renal stones is summarized in Table 7. The number of ESWL sessions had a significant effect on the CMD (p-value = .001), while the adjusted odds ratio (AOR (95% CI)) showed a significance of 0.041. The number of ESWL sessions was 2.47 more likely to disturb the CMD than the normal ones (OR= 2.47, 95% CI= 1.040-6.683), while hydronephrosis was 1.70 more likely to disturb the CMD than the normal ones. Age and number of stones insignificantly affected CMD in this model (Table 7).

**Table 7:** Logistic regression analysis for prediction of CMD disturbance in patients exposed with ESWL for treatment of renal stones Logistic regression analysis for the prediction of CMD disturbance in patients exposed to ESWL for the treatment of renal stones,  related to age, number of stones, frequency of ESWL, and hydronephrosis (p < 0.05, 0.01 considered statistically significant).

Variables	COR (95% CI)	P-value	AOR (95% CI)	P-value
The age	.99 (.970–1.010)	.329	.986 (.966–1.007)	.194
Number of stones	1.30 (.935–1.816)	.118	1.22 (.867–1.726)	.252
Number of ESWL sessions	1.32 (1.126–1.535	.001	2.47 (1.040–6.683)	.041
Hydronephrosis	1.64 (1.154–2.336)	.006	1.70 (1.188–2.434)	.004

## Discussion

Tissue damage to the kidneys may occur due to intensive ESWL. When a focused zone is targeted, such as the renal calyceal system, tissue damage most frequently entails trauma that is mainly restricted to that area. Consequently, these changes may affect kidney length and corticomedullary differentiation (CMD). The relationship between ESWL-induced kidney size and CMD was examined in patients with renal stones. The findings of this study revealed that ESWL reduced kidney length and disturbed CMD in patients with renal stones.

The results of this study indicated that the most common finding in post-lithotripsy patients was hydronephrosis, present in 30.1% of cases, and disturbance in CMD in 19.1% of patients. Kaude et report that in 29% of the patients who underwent ESWL, there were varying degrees of hydronephrosis [17]. Another study reported that the CMD was not preserved in 17.9% of the lithotripsy patients [18]. 

The impact of ESWL on living tissues has been investigated in several studies. The present study found a significant reduction in the renal length and disturbed CMD in patients treated with ESWL. The mean kidney length in the exposed group was significantly lower than that in the control group. Animal studies have shown that SWs harm the vascular system of the kidneys [7, 19-21]. According to a morphological study of pig kidneys treated with a clinical dose of SWL, the veins are particularly vulnerable to damage. Vascular damage affects various vessels from the vasa recta and cortical capillaries to the intralobular and arcuate arteries and veins [7, 19-22]. The findings of these animal studies on SWL injury-approved vascular damage. Since SWL injury is dominated by vascular trauma, it is unsurprising that patients with clotting disorders experience kidney damage, affect kidney length, and disturb CMD. Furthermore, previous studies have supported the evidence of delayed kidney growth in pediatric patients. This delayed growth of the kidneys consequently decreases kidney length [6, 22]. 

Numerous factors affect the severity of kidney injury, which may affect kidney size and reduce the length and CMD. Animal studies have demonstrated that the quantity of SWL delivered, lithotripter power setting, and rate of SW delivery all affect kidney damage in SWL [23-31].

The current study showed that hydronephrosis is a contributing factor to disturbed CMD. A previous study showed that the degree of hydronephrosis caused by stones did not affect the success rate of SWL. However, stones in obstructed systems tend to require repeated treatment and a prolonged time for stone clearance [32]. Kaude et al. consistently studied renal morphology and function immediately after ESWL and found loss of CMD and partial parenchymal obstruction [5]. Latif et al. study involving 50 participants who underwent ESWL, followed for a period of one year (1, 3, 6 months, and 1 year) by ultrasound, CT scan (Computerized Tomography and Intravenous Urography (IVU)) to determine the kidney morphology changes after ESWL, mention that parenchymal scarring and reduced kidney size were identified by ultrasound after ESWL 6, hydronephrosis, obstruction at the pelviureteric junction and ureter, as well as steinstrasse formation following ESWL 3 [33]. The morphologic and functional alterations are attributed to renal contusion, which causes edema and extravasation of urine and blood into the interstitial, subcapsular, and perirenal regions [5]. Therefore, these processes may cause kidney injury, leading to a disturbed CMD and reduced renal length. 

This study has some limitations because it is a cross-sectional study that lacks follow-up of the participants and repetitive assessments of kidney length during the study period. Thus, follow-up sonographic examinations are needed to investigate the effects of ESWL on KL and CMD before and after exposure to SWL. In general, the results were not completely influenced by this factor, as the larger sample size in our study showed significant findings. Further prospective studies are needed to investigate patients' pre-and post-ESWL treatment and to confirm the initial results of this study.

## Conclusions

This study assessed the impact of ESWL on renal morphology, particularly KL and CMD, in patients with renal stones. The majority of renal stones were solitary and predominantly measured between 0.3-1 cm, with most stones located in the lower calyces. A minority of ESWL patients presented with mild to moderate hydronephrosis. Increased number of ESWL sessions for treating renal stones significantly affects renal length and CMD - the renal length was significantly reduced and CMD was distorted in patients exposed to more SWL sessions. The outcomes were not influenced by the number of stones. This study highlights the potential influence of repetitive ESWL sessions on kidney morphology, underscoring the importance of monitoring KL and CMD in patients undergoing treatment. Understanding these effects is critical for improving treatment protocols, minimizing kidney damage, and ensuring better outcomes for patients.
